# Supplementary material for: A comparative study of the cortical function during the interpretation of algorithms in pseudocode and the solution of first-order algebraic equations
Source: PLoS One. 2023 Jun 27;18(6):e0274713. doi: 10.1371/journal.pone.0274713 (PMC10298793; doi:10.1371/journal.pone.0274713)
Supplement: S1 Table — p-values that resulted from the evaluation (asymptotic 2-tailed Mann & Witney U test) of the pairwise task differences on the parameters SWN and Eg. (PDF) [file pone.0274713.s001.pdf]

DELTA BAND											
GP	Task	Mean	SD	p-value							
				ES	*EM*	*EC*	*PS*	*PM*	*PC*	*CO*	*DO*
SWN	***ES***	0.95790	0.03505	—	2.152E-01	8.662E-02	**2.677E-06**	**7.039E-09**	**2.871E-09**	8.653E-01	3.000E-01
	EM	0.96243	0.03491	2.152E-01	—	6.375E-01	**3.782E-04**	**2.373E-06**	**9.016E-07**	9.249E-01	5.591E-01
	EC	0.96425	0.03585	8.662E-02	6.375E-01	—	**1.879E-03**	**1.258E-05**	**3.571E-06**	7.774E-01	6.647E-01
	PS	0.97378	0.03076	**2.677E-06**	**3.782E-04**	**1.879E-03**	—	2.590E-01	2.394E-01	3.179E-01	8.358E-01
	PM	0.97776	0.02731	**7.039E-09**	**2.373E-06**	**1.258E-05**	2.590E-01	—	9.351E-01	1.468E-01	7.487E-01
	PC	0.97861	0.02381	**2.871E-09**	**9.016E-07**	**3.571E-06**	2.394E-01	9.351E-01	—	1.178E-01	7.487E-01
	CO	0.95891	0.03330	8.653E-01	9.249E-01	7.774E-01	3.179E-01	1.468E-01	1.178E-01	—	3.179E-01
	DO	0.96946	0.04094	3.000E-01	5.591E-01	6.647E-01	8.358E-01	7.487E-01	7.487E-01	3.179E-01	—
Eg	***ES***	0.31276	0.06837	—	2.107E-01	8.580E-02	**2.650E-06**	**6.914E-09**	**2.853E-09**	7.487E-01	2.828E-01
	EM	0.30049	0.05638	2.107E-01	—	6.383E-01	**3.776E-04**	**2.363E-06**	**9.016E-07**	1.000E+00	4.624E-01
	EC	0.29696	0.05359	8.580E-02	6.383E-01	—	**1.883E-03**	**1.258E-05**	**3.582E-06**	8.951E-01	6.376E-01
	PS	0.28106	0.04104	**2.650E-06**	**3.776E-04**	**1.883E-03**	—	2.588E-01	2.390E-01	3.365E-01	9.850E-01
	PM	0.27422	0.03175	**6.914E-09**	**2.363E-06**	**1.258E-05**	2.588E-01	—	9.370E-01	1.689E-01	8.065E-01
	PC	0.27197	0.02592	**2.853E-09**	**9.016E-07**	**3.582E-06**	2.390E-01	9.370E-01	—	1.576E-01	8.065E-01
	CO	0.30122	0.05432	7.487E-01	1.000E+00	8.951E-01	3.365E-01	1.689E-01	1.576E-01	—	3.179E-01
	DO	0.29050	0.05865	2.828E-01	4.624E-01	6.376E-01	9.850E-01	8.065E-01	8.065E-01	3.179E-01	—
