# Supplementary material for: A comparative study of the cortical function during the interpretation of algorithms in pseudocode and the solution of first-order algebraic equations
Source: PLoS One. 2023 Jun 27;18(6):e0274713. doi: 10.1371/journal.pone.0274713 (PMC10298793; doi:10.1371/journal.pone.0274713)
Supplement: S2 Table — p-values that resulted from the evaluation (asymptotic 2-tailed Mann & Witney U test) of the pairwise task differences on the parameters SWN and Eg. (PDF) [file pone.0274713.s002.pdf]

THETA BAND											
GP	Task	Mean	SD	p-value							
				ES	*EM*	*EC*	*PS*	*PM*	*PC*	*CO*	*DO*
SWN	*ES*	1.26863	0.04230	—	**2.071E-02**	**1.309E-02**	**4.171E-08**	**2.330E-05**	**3.864E-07**	2.662E-01	**9.832E-03**
	EM	1.25529	0.05380	**2.071E-02**	—	8.979E-01	**3.494E-16**	**9.531E-12**	**1.579E-14**	1.366E-01	**8.809E-03**
	EC	1.25304	0.05936	**1.309E-02**	8.979E-01	—	**4.497E-18**	**2.873E-13**	**2.303E-16**	9.351E-02	**4.988E-03**
	PS	1.29810	0.04226	**4.171E-08**	**3.494E-16**	**4.497E-18**	—	2.115E-01	6.649E-01	7.774E-01	3.758E-01
	PM	1.29041	0.05245	**2.330E-05**	**9.531E-12**	**2.873E-13**	2.115E-01	—	3.941E-01	9.249E-01	2.503E-01
	PC	1.29688	0.04412	**3.864E-07**	**1.579E-14**	**2.303E-16**	6.649E-01	3.941E-01	—	8.653E-01	2.828E-01
	CO	1.28960	0.05867	2.662E-01	1.366E-01	9.351E-02	7.774E-01	9.249E-01	8.653E-01	—	3.000E-01
	DO	1.31816	0.05852	**9.832E-03**	**8.809E-03**	**4.988E-03**	3.758E-01	2.503E-01	2.828E-01	3.000E-01	—
Eg	*ES*	0.12909	0.01173	—	**4.950E-02**	**3.917E-02**	**2.704E-09**	**3.263E-06**	**3.023E-08**	1.689E-01	**5.603E-03**
	EM	0.13173	0.01547	**4.950E-02**	—	9.339E-01	**2.990E-16**	**7.555E-12**	**1.046E-14**	1.269E-01	**7.044E-03**
	EC	0.13248	0.01865	**3.917E-02**	9.339E-01	—	**5.445E-18**	**3.227E-13**	**2.207E-16**	9.351E-02	**3.937E-03**
	PS	0.12064	0.01081	**2.704E-09**	**2.990E-16**	**5.445E-18**	—	2.130E-01	6.910E-01	9.549E-01	3.000E-01
	PM	0.12275	0.01399	**3.263E-06**	**7.555E-12**	**3.227E-13**	2.130E-01	—	3.876E-01	8.653E-01	1.809E-01
	PC	0.12082	0.01033	**3.023E-08**	**1.046E-14**	**2.207E-16**	6.910E-01	3.876E-01	—	8.951E-01	2.351E-01
	CO	0.12250	0.01390	1.689E-01	1.269E-01	9.351E-02	9.549E-01	8.653E-01	8.951E-01	—	3.000E-01
	DO	0.11618	0.01265	**5.603E-03**	**7.044E-03**	**3.937E-03**	3.000E-01	1.809E-01	2.351E-01	3.000E-01	—
