# Supplementary material for: A comparative study of the cortical function during the interpretation of algorithms in pseudocode and the solution of first-order algebraic equations
Source: PLoS One. 2023 Jun 27;18(6):e0274713. doi: 10.1371/journal.pone.0274713 (PMC10298793; doi:10.1371/journal.pone.0274713)
Supplement: S3 Table — p-values that resulted from the evaluation (asymptotic 2-tailed Mann & Witney U test) of the pairwise task differences on the parameters SWN and Eg. (PDF) [file pone.0274713.s003.pdf]

ALPHA BAND											
**GP**	**Task**	**Mean**	**SD**	**p-value**							
				ES	*EM*	*EC*	*PS*	*PM*	*PC*	*CO*	*DO*
**SWN**	***ES***	1.30408	0.03516		**1.655E-05**	**2.902E-05**	**4.770E-07**	**9.161E-06**	**5.435E-05**	1.935E-01	2.828E-01
	EM	1.32275	0.04049	**1.655E-05**		8.551E-01	5.678E-01	9.606E-01	6.586E-01	8.065E-01	8.653E-01
	EC	1.32447	0.04216	**2.902E-05**	8.551E-01		4.267E-01	8.245E-01	8.538E-01	7.203E-01	9.249E-01
	PS	1.32652	0.03325	**4.770E-07**	5.678E-01	4.267E-01		5.019E-01	3.077E-01	8.951E-01	8.951E-01
	PM	1.32499	0.03517	**9.161E-06**	9.606E-01	8.245E-01	5.019E-01		6.832E-01	1.000E+00	9.549E-01
	PC	1.32294	0.03378	**5.435E-05**	6.586E-01	8.538E-01	3.077E-01	6.832E-01		8.951E-01	9.850E-01
	CO	1.32368	0.04081	1.935E-01	8.065E-01	7.203E-01	8.951E-01	1.000E+00	8.951E-01		6.647E-01
	DO	1.31429	0.04640	2.828E-01	8.653E-01	9.249E-01	8.951E-01	9.549E-01	9.850E-01	6.647E-01	
**Eg**	***ES***	0.12038	0.00811		**4.993E-07**	**3.596E-07**	**3.796E-09**	**1.208E-07**	**7.577E-07**	**3.646E-02**	9.351E-02
	EM	0.11538	0.00870	**4.993E-07**		9.968E-01	5.131E-01	9.604E-01	7.824E-01	6.647E-01	8.653E-01
	EC	0.11471	0.00841	**3.596E-07**	9.968E-01		4.899E-01	8.844E-01	8.304E-01	6.647E-01	7.487E-01
	PS	0.11425	0.00656	**3.796E-09**	5.131E-01	4.899E-01		5.164E-01	3.409E-01	1.000E+00	9.549E-01
	PM	0.11456	0.00686	**1.208E-07**	9.604E-01	8.844E-01	5.164E-01		7.272E-01	8.951E-01	8.653E-01
	PC	0.11491	0.00666	**7.577E-07**	7.824E-01	8.304E-01	3.409E-01	7.272E-01		6.376E-01	8.653E-01
	CO	0.11445	0.00853	**3.646E-02**	6.647E-01	6.647E-01	1.000E+00	8.951E-01	6.376E-01		6.647E-01
	DO	0.11660	0.01045	9.351E-02	8.653E-01	7.487E-01	9.549E-01	8.653E-01	8.653E-01	6.647E-01	
