# Supplementary material for: A comparative study of the cortical function during the interpretation of algorithms in pseudocode and the solution of first-order algebraic equations
Source: PLoS One. 2023 Jun 27;18(6):e0274713. doi: 10.1371/journal.pone.0274713 (PMC10298793; doi:10.1371/journal.pone.0274713)
Supplement: S4 Table — p-values that resulted from the evaluation (asymptotic 2-tailed Mann & Witney U test) of the pairwise task differences on the parameters SWN and Eg. (PDF) [file pone.0274713.s004.pdf]

BETA BAND											
GP	Task	Mean	SD	p-value							
				ES	*EM*	*EC*	*PS*	*PM*	*PC*	*CO*	*DO*
SWN	***ES***	1.56601	0.07256	—	7.190E-01	8.848E-01	6.632E-01	5.178E-01	8.389E-01	8.951E-01	4.178E-01
	EM	1.56715	0.07894	7.190E-01	—	5.865E-01	9.347E-01	7.927E-01	7.402E-01	9.249E-01	6.109E-01
	EC	1.56294	0.07885	8.848E-01	5.865E-01	—	5.480E-01	4.399E-01	9.188E-01	9.850E-01	4.856E-01
	PS	1.56500	0.08216	6.632E-01	9.347E-01	5.480E-01	—	8.357E-01	6.752E-01	8.653E-01	6.647E-01
	PM	1.56281	0.09884	5.178E-01	7.927E-01	4.399E-01	8.357E-01	—	6.484E-01	9.549E-01	6.923E-01
	PC	1.56079	0.09277	8.389E-01	7.402E-01	9.188E-01	6.752E-01	6.484E-01	—	8.653E-01	4.624E-01
	CO	1.56859	0.07581	8.951E-01	9.249E-01	9.850E-01	8.653E-01	9.549E-01	8.653E-01	—	4.856E-01
	DO	1.58416	0.07201	4.178E-01	6.109E-01	4.856E-01	6.647E-01	6.923E-01	4.624E-01	4.856E-01	—
Eg	***ES***	0.08020	0.00850	—	2.254E-01	3.602E-01	1.877E-01	1.196E-01	2.259E-01	5.847E-01	3.000E-01
	EM	0.07951	0.00921	2.254E-01	—	7.227E-01	9.225E-01	7.605E-01	8.516E-01	1.000E+00	5.340E-01
	EC	0.07978	0.00948	3.602E-01	7.227E-01	—	6.782E-01	5.347E-01	9.835E-01	9.850E-01	4.856E-01
	PS	0.07980	0.01033	1.877E-01	9.225E-01	6.782E-01	—	8.061E-01	7.441E-01	9.850E-01	5.340E-01
	PM	0.08061	0.01404	1.196E-01	7.605E-01	5.347E-01	8.061E-01	—	6.955E-01	9.850E-01	6.376E-01
	PC	0.08062	0.01318	2.259E-01	8.516E-01	9.835E-01	7.441E-01	6.955E-01	—	9.549E-01	3.964E-01
	CO	0.07885	0.00829	5.847E-01	1.000E+00	9.850E-01	9.850E-01	9.850E-01	9.549E-01	—	5.095E-01
	DO	0.07714	0.00757	3.000E-01	5.340E-01	4.856E-01	5.340E-01	6.376E-01	3.964E-01	5.095E-01	—
