# Supplementary material for: A comparative study of the cortical function during the interpretation of algorithms in pseudocode and the solution of first-order algebraic equations
Source: PLoS One. 2023 Jun 27;18(6):e0274713. doi: 10.1371/journal.pone.0274713 (PMC10298793; doi:10.1371/journal.pone.0274713)
Supplement: S5 Table — p-values that resulted from the evaluation (asymptotic 2-tailed Mann & Witney U test) of the pairwise task differences on the parameters SWN and Eg. (PDF) [file pone.0274713.s005.pdf]

GAMMA BAND											
GP	Task	Mean	SD	p-value							
				ES	*EM*	*EC*	*PS*	*PM*	*PC*	*CO*	*DO*
SWN	***ES***	1.44649	0.11274	—	4.534E-01	4.741E-01	1.973E-01	6.952E-01	4.055E-01	3.758E-01	8.358E-01
	EM	1.44193	0.12902	4.534E-01	—	9.356E-01	6.663E-01	7.618E-01	9.683E-01	5.095E-01	8.065E-01
	EC	1.43878	0.12273	4.741E-01	9.356E-01	—	5.416E-01	9.081E-01	7.043E-01	4.178E-01	9.249E-01
	PS	1.43367	0.11896	1.973E-01	6.663E-01	5.416E-01	—	5.340E-01	9.976E-01	5.095E-01	9.850E-01
	PM	1.43642	0.13572	6.952E-01	7.618E-01	9.081E-01	5.340E-01	—	5.938E-01	3.964E-01	8.951E-01
	PC	1.43443	0.12879	4.055E-01	9.683E-01	7.043E-01	9.976E-01	5.938E-01	—	5.591E-01	1.000E+00
	CO	1.41402	0.10191	3.758E-01	5.095E-01	4.178E-01	5.095E-01	3.964E-01	5.591E-01	—	6.376E-01
	DO	1.43598	0.11109	8.358E-01	8.065E-01	9.249E-01	9.850E-01	8.951E-01	1.000E+00	6.376E-01	—
Eg	***ES***	0.09686	0.01816	—	6.945E-01	7.499E-01	3.567E-01	9.446E-01	6.349E-01	4.856E-01	8.951E-01
	EM	0.09761	0.02020	6.945E-01	—	9.666E-01	6.558E-01	7.681E-01	9.541E-01	5.591E-01	9.249E-01
	EC	0.09795	0.02100	7.499E-01	9.666E-01	—	4.985E-01	9.577E-01	6.715E-01	4.856E-01	9.249E-01
	PS	0.09879	0.02168	3.567E-01	6.558E-01	4.985E-01	—	5.429E-01	9.833E-01	5.340E-01	9.249E-01
	PM	0.10006	0.02952	9.446E-01	7.681E-01	9.577E-01	5.429E-01	—	6.100E-01	4.856E-01	1.000E+00
	PC	0.09995	0.02813	6.349E-01	9.541E-01	6.715E-01	9.833E-01	6.100E-01	—	5.847E-01	9.850E-01
	CO	0.10031	0.01684	4.856E-01	5.591E-01	4.856E-01	5.340E-01	4.856E-01	5.847E-01	—	6.109E-01
	DO	0.09727	0.01770	8.951E-01	9.249E-01	9.249E-01	9.249E-01	1.000E+00	9.850E-01	6.109E-01	—
