# Supplementary material for: A comparative study of the cortical function during the interpretation of algorithms in pseudocode and the solution of first-order algebraic equations
Source: PLoS One. 2023 Jun 27;18(6):e0274713. doi: 10.1371/journal.pone.0274713 (PMC10298793; doi:10.1371/journal.pone.0274713)
Supplement: S6 Table — (PDF) [file pone.0274713.s006.pdf]

Network index	***Task***	***Delta band***	***Theta band***	***Alpha band***	***Beta band***	***Gamma band***
SWN	Math	3.560E-04	2.880E-03	2.667E-06	4.438E-02	4.763E-01
	Prog.	5.781E-03	1.123E-01	4.079E-01	9.445E-01	8.365E-01
EG	Math	1.223E-04	9.296E-03	1.065E-09	7.285E-03	8.580E-01
	Prog.	5.781E-03	8.354E-02	4.550E-01	9.097E-01	8.187E-01
