# Supplementary figures and images for: A comparative study of the cortical function during the interpretation of algorithms in pseudocode and the solution of first-order algebraic equations
Source: PLoS One. 2023 Jun 27;18(6):e0274713. doi: 10.1371/journal.pone.0274713 (PMC10298793; doi:10.1371/journal.pone.0274713)

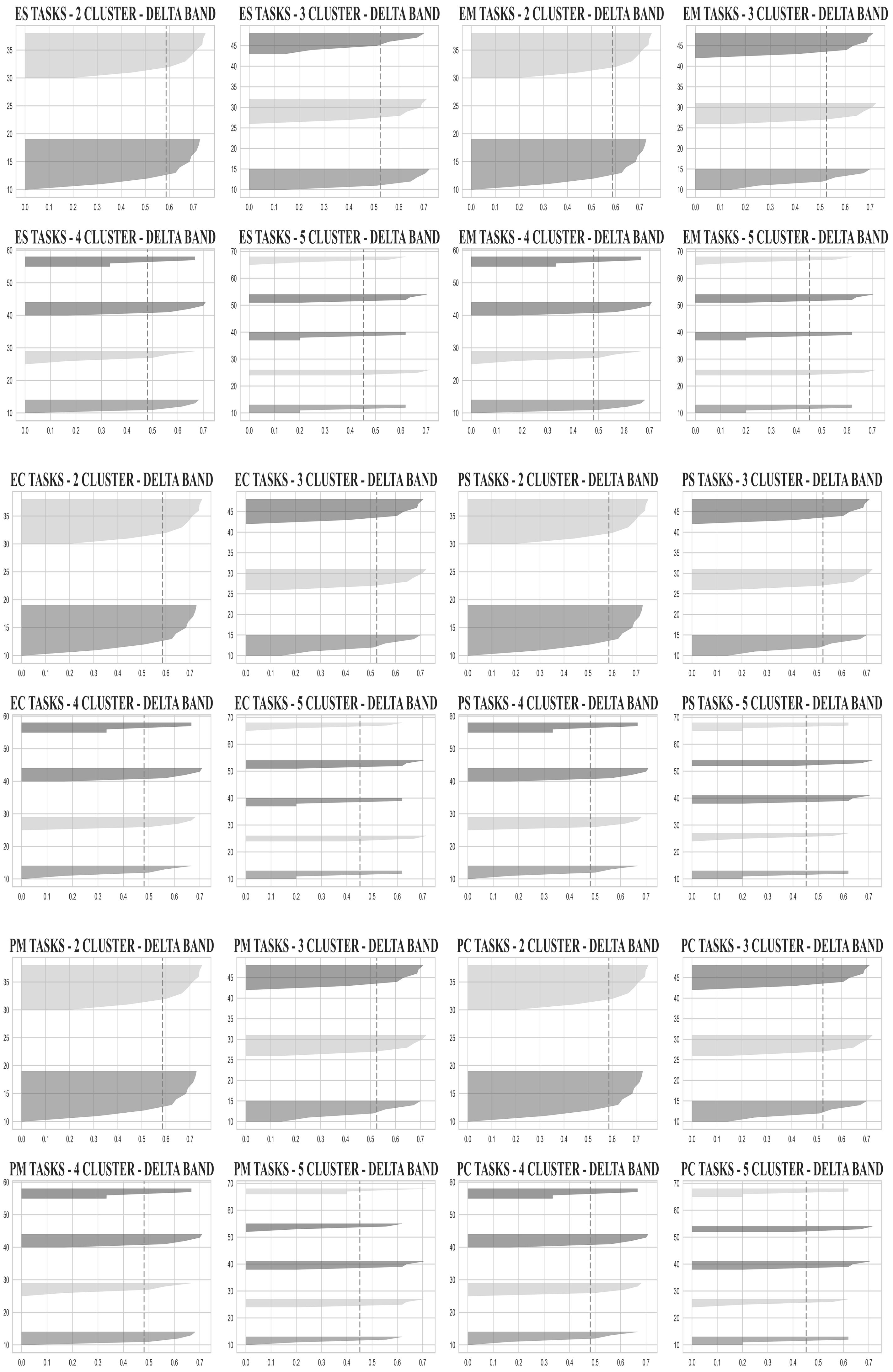

Supplement: S1 Fig — Silhouette plots to select the cluster number on KMeans clustering for local efficiencies on delta band. (TIF) [file pone.0274713.s007.tif]

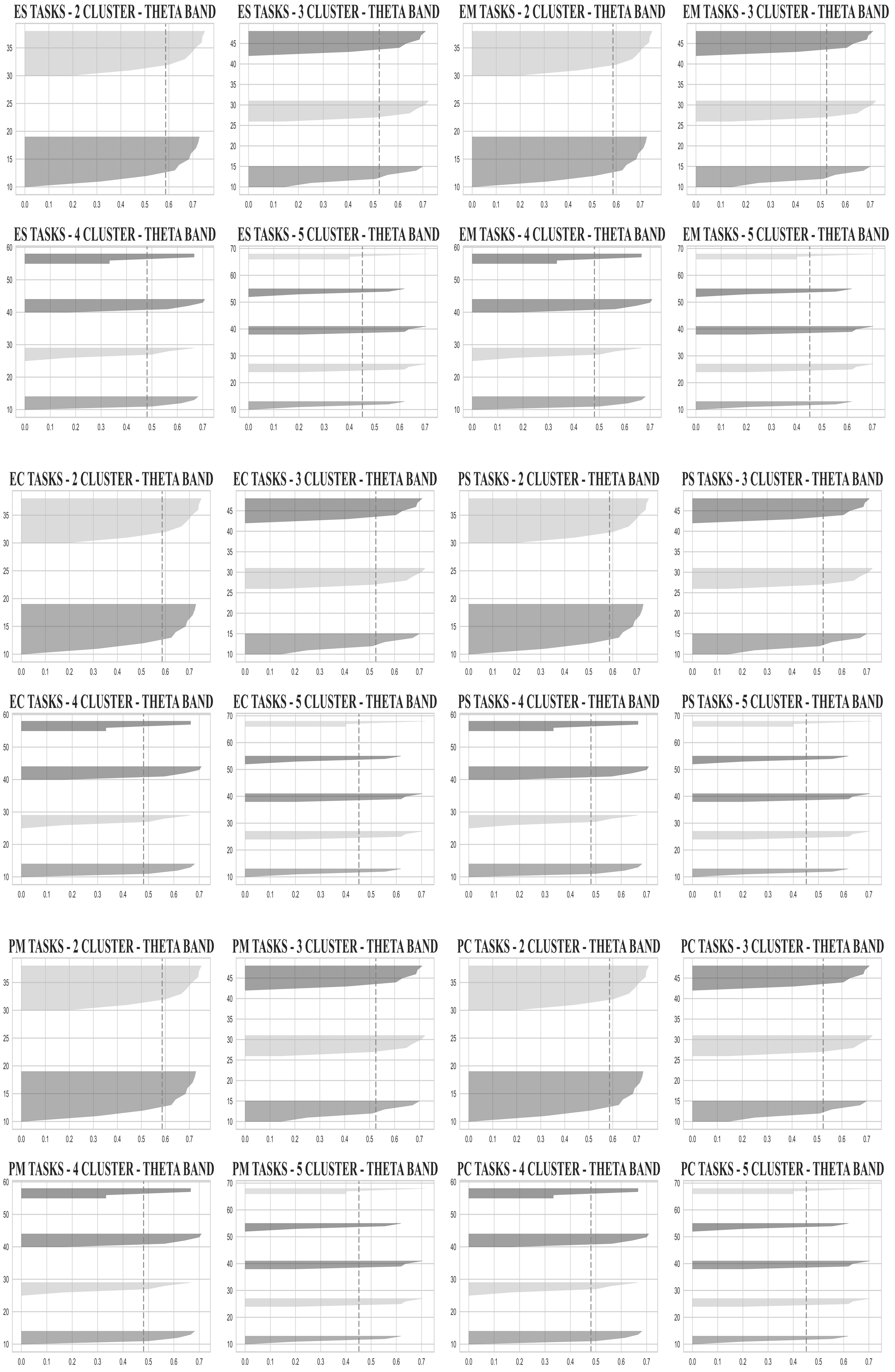

Supplement: S2 Fig — Silhouette plots to select the cluster number on KMeans clustering for local efficiencies on theta band .png. (TIF) [file pone.0274713.s008.tif]

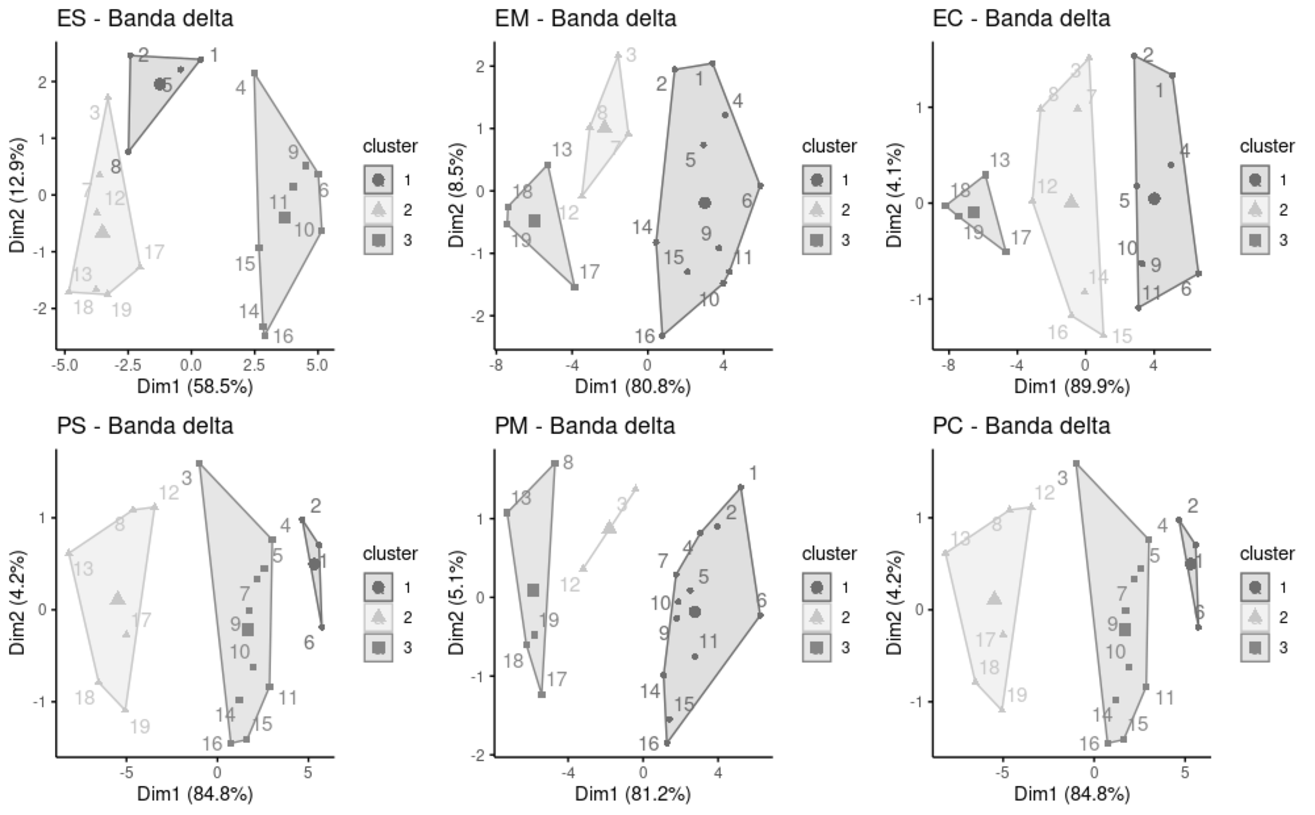

Supplement: S3 Fig — Direct results of applying K-means clustering on the delta band. Fig 6 was obtained from these results. (TIF) [file pone.0274713.s009.tif]

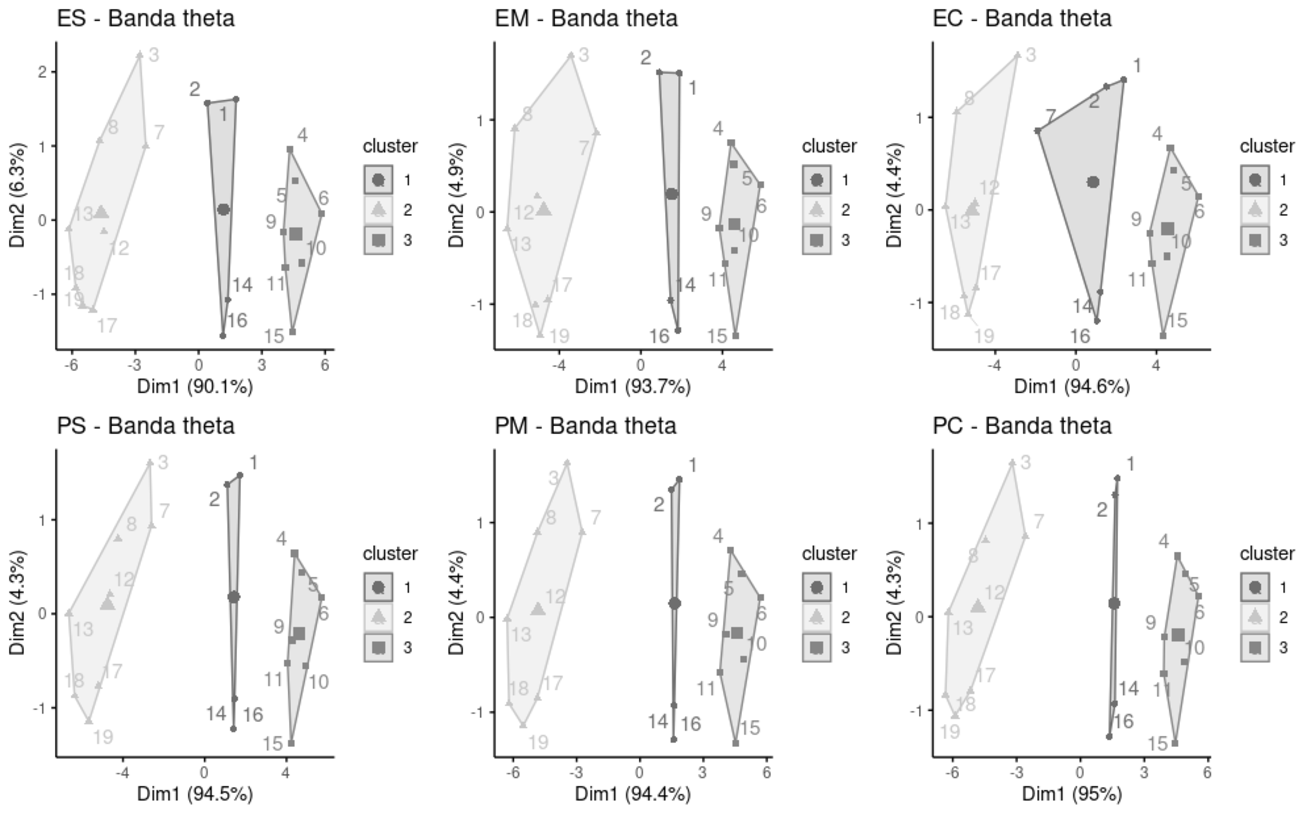

Supplement: S4 Fig — Direct results of applying K-means clustering on the theta band. Fig 7 was obtained from these results. (TIF) [file pone.0274713.s010.tif]
