# Supplementary material for: A comparative study of the cortical function during the interpretation of algorithms in pseudocode and the solution of first-order algebraic equations
Source: PLoS One. 2023 Jun 27;18(6):e0274713. doi: 10.1371/journal.pone.0274713 (PMC10298793; doi:10.1371/journal.pone.0274713)
Supplement: Attachment — Submitted filename: Review on PLOS one [PONE-D-22-24455].docx [file pone.0274713.s011.docx]

Review on PLOS one [PONE-D-22-24455]

A comparative study of the cortical function during the interpretation of algorithms in pseudocode and the solution of first-order algebraic equations

**Decision: Major revision**

# Motivation, novelty and conclusion

This submission has a clear and strong motivation to understand the cortical function difference, using EEG, during mathematical and computational challenges. The novelty of the paper is clear and I am happy that the conclusion can be made via a clearly designed empirical study and a relatively solid statistical analysis.

# Metrics, and research methodology

1. The demonstration of design, shown in Figure 1, is very clear. A minor note, I am very curious about how the order of challenging matters for the final conclusion (e.g., will some level of task randomization influence the final conclusion).
2. I am happy to see researchers apply solid graphical analysis via metrics to define and interpret their conclusions and findings. A few minor notes:
   a. I understand the SWN is a very useful metric to understand connectivity. However, it might be very easy to understand for most readers. Maybe we can demonstrate a few examples (either real data examples, or toy examples) to show a better visualization.
   b. SWN is known to be very sensitive to network size. This might need further clarification. A similar metric can be L_r/L - C / C_l where r is for randomized graph and l is for lattice graph.
   c. The definition of C_rand is not very clear – is it generated through Erdos-Renyi with a fixed number of nodes/edges or with respect to some random weighting?
   d. Eg seems to have the same definition of C?
   e. I am not suggesting removing the discussion of Eg and El, but it seems to be the SWN analysis is more interesting.

# Statistical analysis and result

This submission uses solid statistical analysis methods to support the majority of their findings, which are mostly innovative and convincing. Some major or minor comments below.

1. [Major] It is not clarified in the paper how replication was performed for each sample. As there are only 16 subjects enrolled in this study, it would be helpful to understand whether the sample replication was performed and so how the sample-level heterogeneity/fixed effect influenced the research conclusion.
2. [Major] Authors applied pairwise non-parametric tests as evidence to show their conclusion. I am curious to understand how the multiple group anova or mixed effect generalized linear model tells us. For example, the difficulty can be a categorical variable, band can be another covariate, computing/mathematical task can be another dummy variable. A few interactions can also be useful (e.g., interaction between problem types and difficulties, etc). This can help us to gather more information related to interactions, rather than the pairwise comparison.
3. [Major] In the supplementary table S1 - S5, which supports the main conclusion of the paper, it includes a large amount of p-values. There is a concern of multiplicity (e.g., false positive with multiple tests). Those p-values less than 0.05 (highlighted) are very tiny (e.g., <10^-5) so I am supportive of most of the key findings but it is worth checking or at least clarifying how the result will change if we apply multiplicity adjustment.
4. [Minor] F3 and F4 are informative but there might be a few ways to improve. E.g., 1 is a relatively important cutoff for small world parameters, so it would be helpful to align with the same y axes range, and to clarify/interpret the result.
5. [Minor] I am not seeing an informative interpretation on Fig 5. However, as you find some significant differences in SWN, I am very keen to see a few demonstrations or toy examples showing how visually the connectivity differs.
